# Targeting MALT1 Suppresses the Malignant Progression of Colorectal Cancer *via* miR-375/miR-365a-3p/NF-κB Axis

**DOI:** 10.3389/fcell.2022.845048

**Published:** 2022-03-02

**Authors:** Rui Qian, Xinli Niu, Yinghui Wang, Zhi Guo, Xuyi Deng, Zhenhua Ding, Meijuan Zhou, Haijun Deng

**Affiliations:** ^1^ Department of General Surgery and Guangdong Provincial Key Laboratory of Precision Medicine for Gastrointestinal Tumor, Nanfang Hospital, The First School of Clinical Medicine, Southern Medical University, Guangzhou, China; ^2^ Department of Radiation Medicine, Guangdong Provincial Key Laboratory of Tropical Disease Research, School of Public Health, Southern Medical University, Guangzhou, China; ^3^ Jiangmen Central Hospital, Affiliated Jiangmen Hospital of Sun Yat-sen University, Jiangmen, China

**Keywords:** MALT1, CRC, NF-κB, MI-2, miRNA

## Abstract

Colorectal cancer (CRC) is a malignant tumor with the second highest morbidity and the third highest mortality in the world, while the therapeutic options of targeted agents remain limited. Here, mucosa-associated lymphoid tissue lymphoma translocation protein 1 (MALT1), known as the upstream of the NF-κB signaling pathway, was identified to be highly upregulated in CRC tumors and cell lines. Furthermore, the downregulation of MALT1 or inhibition of its proteolytic function by MI-2 suppressed the cell proliferation and migration of CRC cells. *In vivo*, suppressing the MALT1 expression or its proteasome activity effectively reduced the size of the subcutaneous tumor in nude mice. Mechanistically, miR-375 and miR-365a-3p were identified to inhibit NF-κB activation *via* targeting MALT1. Overall, our results highlight that a novel regulatory axis, miRNA-MALT1-NF-κB, plays a vital role in the progression of CRC and provides novel and hopeful therapeutic targets for clinical treatment.

## Introduction

Colorectal cancer (CRC) is the third most common cancer and the second leading cause of cancer-related deaths worldwide. In 2020, there were surprisingly about 1.9 million new cases of CRC and more than 935,000 deaths all over the world. Moreover, the incidence rate of early-onset CRC is rising by 1%–4% per year (Sung et al., 2021), which is paralleled by the increasing mortality rate (Siegel et al., 2020). However, there is still a shortage of effective treatments in clinics. Traditional therapies, like endoscopic treatment and surgery, are limited for local and early-stage patients. Emerging immunotherapies still have not shown an increase of overall survival compared to chemotherapy alone (Dekker et al., 2019). Thus, it is necessary and imperative to put forward new treatment strategies to improve the outcomes of CRC and reduce its increasing mortality.

The NF-κB pathway has a broad role in various cellular responses involving immunity, inflammation, and cancer. Its activation is closely associated with all cancer hallmarks, especially in enhancing cell proliferation and metastasis. Targeting inhibitors, such as proteasome inhibitors and non-steroidal anti-inflammatory drugs (NSAIDs), have been applied in CRC for chemotherapeutic resistance and the prevention of recurrence (Richardson et al., 2003; Patel et al., 2018), whereas they are accompanied by inevitable side events due to their extensive effects (Katona and Weiss, 2020). Thus, it is supposed that target therapies with strong specificity would represent a safer and more beneficial efficacy. MALT1, mucosa-associated lymphoid tissue lymphoma translocation protein 1, is known as a key upstream molecule in the NF-κB pathway, which was identified as a human paracaspase. Its deficiency causes infantile combined immunodeficiency and immune dysregulation (Punwani et al., 2015). In almost all different types of lymphoma, MALT1 is required to cleave NF-κB negative regulators A20 and RelB (Coornaert et al., 2008), while it is less studied in solid tumors (Fontan et al., 2012; Di Pilato et al., 2019; Liu et al., 2020).

MicroRNAs (miRNAs) are a kind of small non-coding RNAs with 18–25 nucleotides in length. They function as post-transcriptional repressors by binding to the 3’ untranslated region (UTR) of target mRNAs. In various types of cancers, miRNA expressions are altered to promote or suppress tumorigenesis and tumor development (Lin and Gregory, 2015). So far, there are several clinical trials of miRNA drugs undergoing to expect an application for tumors. MRX34, a liposomal miR-34a mimic, reached phase-I clinical trials showing that one of three CRC patients had a stable disease (Beg et al., 2017). The ongoing multicenter phase-I clinical trials of miR-16 mimic already showed a 68% stable disease in NSCLC treatment (van Zandwijk et al., 2017). Following the recent advances in miRNA chemistry and delivery technologies, the miRNA-based agents moving into the clinic become more available and a deeper exploration of miRNA drugs in CRC is more imperative (Rupaimoole and Slack, 2017).

In this study, we discovered that MALT1 was highly expressed in CRC and promoted its malignant progression. Our study revealed that downregulated MALT1 or the inhibition of its protease activity with MI-2 inhibited cell proliferation and migration through activating the NF-κB pathway. Meanwhile, miR-365a-3p and miR-375 were identified as the upstream regulators of MALT1. Taken together, these results demonstrated that MALT1 acted as an oncogene in CRC, which would be a promising therapeutic target *via* epigenetic regulation or its activity.

## Methods and Materials

### Patients and Tissue Samples

The CRC tissue samples were obtained from 58 patients who were diagnosed and underwent surgery from January 2010 to December 2011 in Jiangmen Central Hospital, Affiliated Jiangmen Hospital of Sun Yat-sen University. A written informed consent to use the surgical samples has been signed by all the patients, and the study was approved by the Institutional Review Board of Nanfang Hospital affiliated to Southern Medical University. Collected samples were fixed by paraformaldehyde and embedded in paraffin.

### Immunohistochemistry Assay

Paraffin-embedded tissue was cut into 5 μm thick slices, dewaxed with xylene, and hydrated with ethanol. Then, the slides were heated at 95°C in a 0.01 M citrate buffer (*pH* = 6.0) and quenched for peroxidase activity with 3% hydrogen peroxide for 20 min to retrieve antigens. After being treated with 10% goat serum, the slides were incubated overnight with antibodies at 4°C. PBS washing was followed by incubation with goat anti-rabbit IgG for 1 h and then stained with 3,3-diaminobenzidine. When all sections were dehydrated and sealed, we selected images with an inverted microscope (ZEISS). Immunohistochemistry was performed with the following antibodies: Ki67 (27309-1-AP; Proteintech), MALT1 (66225-1-Ig; Proteintech), p-p65 (AB11014, phosphor-Ser536), fibronectin (66042-1-Ig; Proteintech), N-cadherin (22018-1-AP; Proteintech), E-cadherin (20874-1-AP; Proteintech), vimentin (10366-1-AP; Proteintech) Snail1 (26183-1-AP; Proteintech), and Snail2 (12129-1-AP; Proteintech).

### Bioinformatics Analysis

The mRNA microarray datasets (GSE21510, GSE17536) and miRNA microarray datasets (GSE38389, GSE41655, GSE30454, GSE18392) were obtained from the Gene Expression Omnibus (GEO) database (http://www.ncbi.nlm.nih.gov/geo/). In GSE21510, patients were divided into the normal group and tumor group according to the type of tissue resected in the surgeries. The expression of MALT1 is compared by the content of corresponding mRNA. Meanwhile, in GSE17536, patients were divided into high expression group (85%) and low expression group (15%) depending on the protein expression of MALT1, so that we were able to judge the relationship between MALT1 and prognosis by comparing the time of disease-free survival.

### Cell Lines and Culture Conditions

The human CRC cell lines HCT116 and SW480 and the human normal colorectal epithelial cell line NCM460 were purchased from American Type Culture Collection (ATCC, Manassas, VA, United States). All cell lines were cultured in Dulbecco’s modified eagle medium (DMEM; Life Technologies) supplemented with 10% fetal bovine serum (FBS; Beyotime Biotechnology, Shanghai, China), 100 μg/ml streptomycin, and 100 units/ml penicillin. They were all placed in a humidified incubator with 5% CO_2_ at 37°C and a mild atmosphere.

### Reverse Transcription and qPCR

Total RNA was extracted with TRIzol Reagent (ambin, United States). After gDNA was removed, 500 ng of total RNA was reversed with Evo M-MLV RT Kit with gDNA Clean for qPCR II AG11711 (Accurate Biology) according to the manufacturer’s instructions. Real-time PCR was performed using PerfectStart Green qPCR SuperMix (TransGen Biotech) on a LightCycler 96 Detection System (Roche). GAPDH was served as an internal reference gene, and the primers used for qPCR in this study were as follows: MALT1: F, TGG​AAG​CCC​TAT​TCC​TCA​CTA​CC; R, CAT​GAC​ACC​AG-TAG​GTT​CCT​TGG, GAPDH: F, GAT​ATT​GTT​GCC​ATC​AAT​GAC​C; R, AGC​C-TTC​TCC​ATG​GTG​GTG​AAG​A, miR-218-5p: F, TTG​TGC​TTG​ATC​TAA​CCA​TGT; miR-338-3p: F, TCC​AGC​ATC​AGT​GAT​TTT​GTT​G; miR-365-3p: F, TAA​TG-CCC​CTA​AAA​ATC​CTT​AT; miR-375-3p: F, TTT​GTT​CGT​TCG​GCT​CGC​GTG​A; and U6: F, CGT​TCA​CGA​ATT​TGC​GTG​TCA​T. The reverse primer used in the qPCR of miRNA was the mRQ 3′ primer supplied with the microRNA first-strand synthesis and miRNA quantitation kits (Takara).

### Western Blot

Total protein was extracted using a cell lysis buffer (Beyotime, Shanghai, China) mixed with a protease inhibitor cocktail on ice, and protein concentration was determined by the Bradford method. SDS-polyacrylamide gel electrophoresis was use to separate the desired proteins. Subsequently, proteins were transferred to PVDE membranes and blocked with 10% milk powder solution. Membranes were placed in a primary antibody overnight at 4°C and then incubated with a secondary antibody at room temperature for 2 h. Immunoblotting was performed with the following antibodies: *β*-actin (47,778; Santa Cruz Biotechnology), GAPDH (47,724; Santa Cruz Biotechnology), p-p65 (AB11014, phosphor-Ser536), MALT1 (2494S; Cell Signaling Technology), A20 (WL00820; Wanleibio), RELB (WL02922; Wanleibio), Fibronectin (66042-1-Ig; Proteintech), N-cadherin (WL01047; Wanleibio), E-cadherin (20874-1-AP, Proteintech), Vimentin (10366-1-AP; Proteintech) Snail (WL01863; Wanleibio), secondary antibodies’ anti-mouse IgG-HRP (VC297958; Invitrogen), and anti-rabbit IgG-HRP (VC297287; Invitrogen). The data were acquired by Tanon 5200 system (Tanon Science) with Luminata Forte Western HRP substrate (Millipore).

### Cell Viability and Colony Formation Assays

HCT116 and SW480 were seeded into six-well plates at a number of 1 × 10^6^ cells per well, and the cells were transfected with plasmid (MALT1-pcDNA3.1-3xFlag-C; Hunan Fenghui Biotechnology Co.) and siRNA for 6 h. After transfection, cells were seeded into 96-well plates at 1,000 cells per well. CCK-8 was added to each well according to the instructions, and after waiting for 2 h of reaction, the OD value at 450 nm was measured with a microplate reader.

For the colony formation assay, transfected HCT116 were seeded into 6-well plates at 500 cells per well and transfected SW480 were seeded into 6-well plates at 500 cells per well. All cells were cultured in DMEM supplemented with 10% FBS for 14 days. After 14 days, the colonies were fixed with 3.7% formaldehyde permeabilized with pure methanol and stained with 0.1% crystal violet. The ability of colony formation was determined by counting the number of stained colonies.

The siRNA (RIBOBIO) used in this study were as follows: siMALT1_1: sense, CCGGAGAUAAUAAUGUGUGdTdT; antisense, CACACAUUAUUAUCUCCG-GdTdT, siMALT1_2: sense, CUACGAUGAUACCAUUCCAdTdT; and antisense, UG-GAAUGGUAUCAUCGUAGdTdT. The miRNA mimic and inhibitor (RIBOBIO) used in this study were as follows: miRNA-365-3p mimic: sense, AGG​GAC​UUU​UGG​GGG​CA-GAU​GUG; antisense, CAC​AUC​UGC​CCC​CAA​AAG​UCC​CU, miRNA-365-3p inhibitor: sense, CAC​AUC​UGC​CCC​CAA​AAG​UCC​CU, miRNA-375-3p mimic: sense, UUU​GUU​CGU​UCG​GCU​CGC​GUG​A; and antisense, UCA​CGC​GAG​CCG​AAC​G-AAC​AAA; miRNA-375-3p inhibitor: sense, UCA​CGC​GAG​CCG​AAC​GAA​CAA​A.

### Cell Migration Assay

HCT116 and SW480 cells (2.5 × 10^6^) transfected with MALT1 or siMALT1 were seeded to the top migration chambers (Millipore) with 200 μl FBS-free DMEM, and 750 μl of DMEM with 10% FBS was added to the lower chamber. After 30 h, the upper chambers were washed with PBS, fixed with 3.7% formaldehyde, permeabilized with pure methanol, and stained with 0.1% crystal violet. Photographs were recorded under an inverted microscope (ZEISS), and the number of cells was then counted with ImageJ.

### Immunofluorescence Assay

For the immunocytochemistry assay, transfected HCT116 were seeded on glass coverslips in 12-well plates at 2.4 × 10^5^ cells per well. About 24 h later, the cells on glass coverslips were washed by PBS twice, fixed with 4% paraformaldehyde for 30 min, and then permeabilized by 0.5% NP-40 for 20 min at room temperature. After being blocked with 3% BSA (bovine serum albumin; Biotopped), glass coverslips were placed in primary antibody dissolved in 3% BSA overnight at 4°C and then incubated for 1 h with specific fluorescence-conjugated secondary antibodies in the dark. 4′,6-diamidino-2-phenylindole was used to stain the cells on glass coverslips. Photographs were collected under an inverted microscope (ZEISS).

### Dual-Luciferase Reporter Assay

HCT116 and SW480 (8 × 10^4^) was seeded into 24-well plates, co-transfected with NF-κB-Luc reporter vector (Beyotime Biotechnology, Shanghai, China), MALT1 and siMALT1. About 48 h later, luciferase intensity was measured using a dual-luciferase reporter assay kit (Beyotime Biotechnology, Shanghai, China), and the expression of each component was analyzed according to the instructions.

### Subcutaneous Xenograft Model

For the xenograft experiments, BALB/c nude mice (6 weeks old, female) were adopted from Guangdong Medical Laboratory Animal Center. As mentioned earlier, HCT116 cells (5 × 10^6^ cells in 50 μl DMEM) transfected with siNC or siMALT1 were subcutaneously inoculated into the scapular region of nude mice. The body weight and tumor volume [largest diameter × (smallest diameter)^2^/2] were measured daily, and after 7 days, a mixture of DMEM (50 μl) with siNC or siMALT1 (12 μl) and LIPO 2000 (12 μl) was injected into the tumor location every 2 days. Tumors were removed immediately after the mice were sacrificed on Day 21 and frozen at −80°C to prepare for the following experiments.

For the *in vivo* suppression studies of MI-2, HCT116 cells (5 × 10^6^ cells in 50 µl DMEM) were subcutaneously inoculated into the scapular region of nude mice (6 weeks old, male). Three days later, MI-2 and dimethyl sulfoxide (DMSO) dissolved in PBS were injected intraperitoneally at a dose of 25 mg/kg body weight in a volume of 10 μl/g body weight every 2 days. Mice were killed and the tumors were removed after 14 days. All procedures were approved by the Southern Medical University Animal Care and Use Committee. All animal studies were conducted in accordance with institutional guidelines.

### Statistical Analysis

The experimental results were expressed as mean values ± SD. A two-tailed Student’s *t*-test, paired *t*-test, log-rank test, and one-way ANOVA were used for the analysis of statistical difference between two groups in IBM SPSS statistics 25. *p* < 0.05 was considered statistically significant.

## Results

### High Expression of MALT1 Is Associated With CRC Poor Prognosis

**FIGURE 1:**
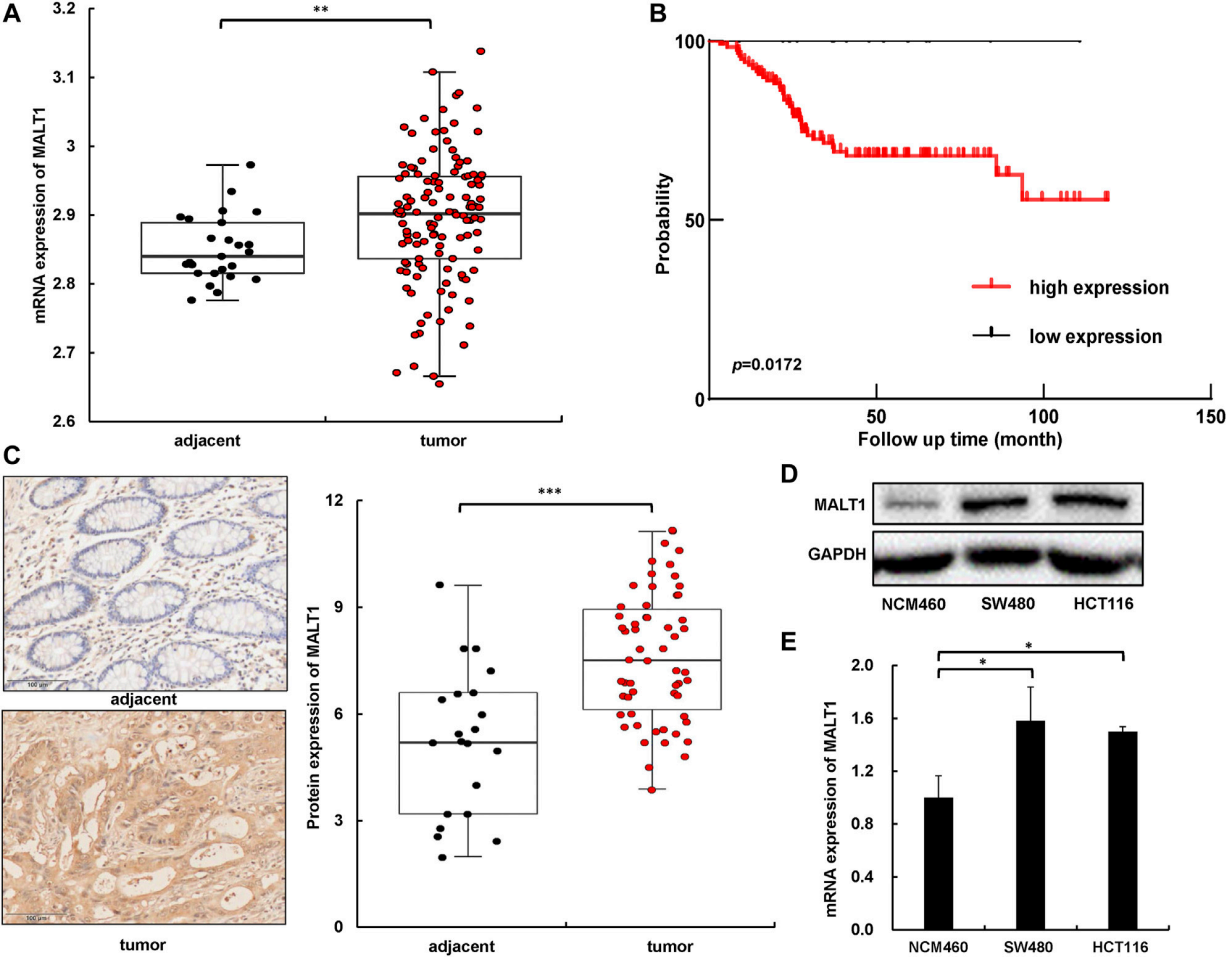
MALT1 was highly expressed in CRC cells and tissues. **(A)** The mRNA level of MALT1 in colorectal tissues from CRC patients was higher than that in adjacent tissues. **(B)** Disease-free survival Kaplan–Meier curves of CRC patients based on expression of MALT1 level. *p*-values were calculated using log-rank test. **(C)** Representative IHC images of MALT1 in tissue specimens. Quantification of MALT1 levels according to IHC scores in adjacent and tumor tissue, respectively (right panel). **(D)** The protein level of MALT1 determined by immunoblotting. **(E)** The mRNA level of MALT1 determined by qPCR. Each experiment was performed in triplicate, and data are presented as mean ± SD. One-way ANOVA and Dunnett’s multiple comparison test were used to analyze the data (*p < 0.05, **p < 0.01, ***p < 0.001).

To explore the relationship between MALT1 and the malignant progression of CRC, we first analyzed the difference of the MALT1 mRNA level between CRC and adjacent tissues based on the Gene Expression Omnibus (GEO) database (GSE21510). The data demonstrated that MALT1 expression was significantly increased in CRC tissues (*n* = 123) compared with adjacent tissues (*n* = 25) (Figure 1A). Another GEO series GSE17536 showed that patients with high MALT1 expression (*n* = 22) had a worse prognosis compared with those with low MALT expression (*n* = 123) (Figure 1B), which reminded us of the significance of MALT1 in CRC. To further confirm the expression of MALT1 in CRC, we examined the protein level of MALT1 by immunohistochemistry (IHC) staining in colorectal tissues from 58 CRC patients compared to adjacent tissues. It was observed by microscope that MALT1 was mainly expressed in tumor tissues, and the IHC scores of tumor tissues were significantly higher than those in adjacent tissues (*p* < 0.001) (Figure 1C). The protein and mRNA levels of MALT1 in CRC lines (SW480, HCT116) and a normal colorectal mucosa cell line (NCM) were detected by immunoblotting and qPCR. Consistent with the results from IHC, the protein and mRNA levels of MALT1 in the SW480 and HCT116 cells were both higher than those in NCM (Figures 1D,E). In light of these findings described, it is concluded that MALT1 was highly expressed in CRC cells and tissues, and we speculated that MALT1 may be associated with the malignancy of CRC.

### Downregulation of MALT1 Inhibits the Proliferation of CRC Cells

**FIGURE 2:**
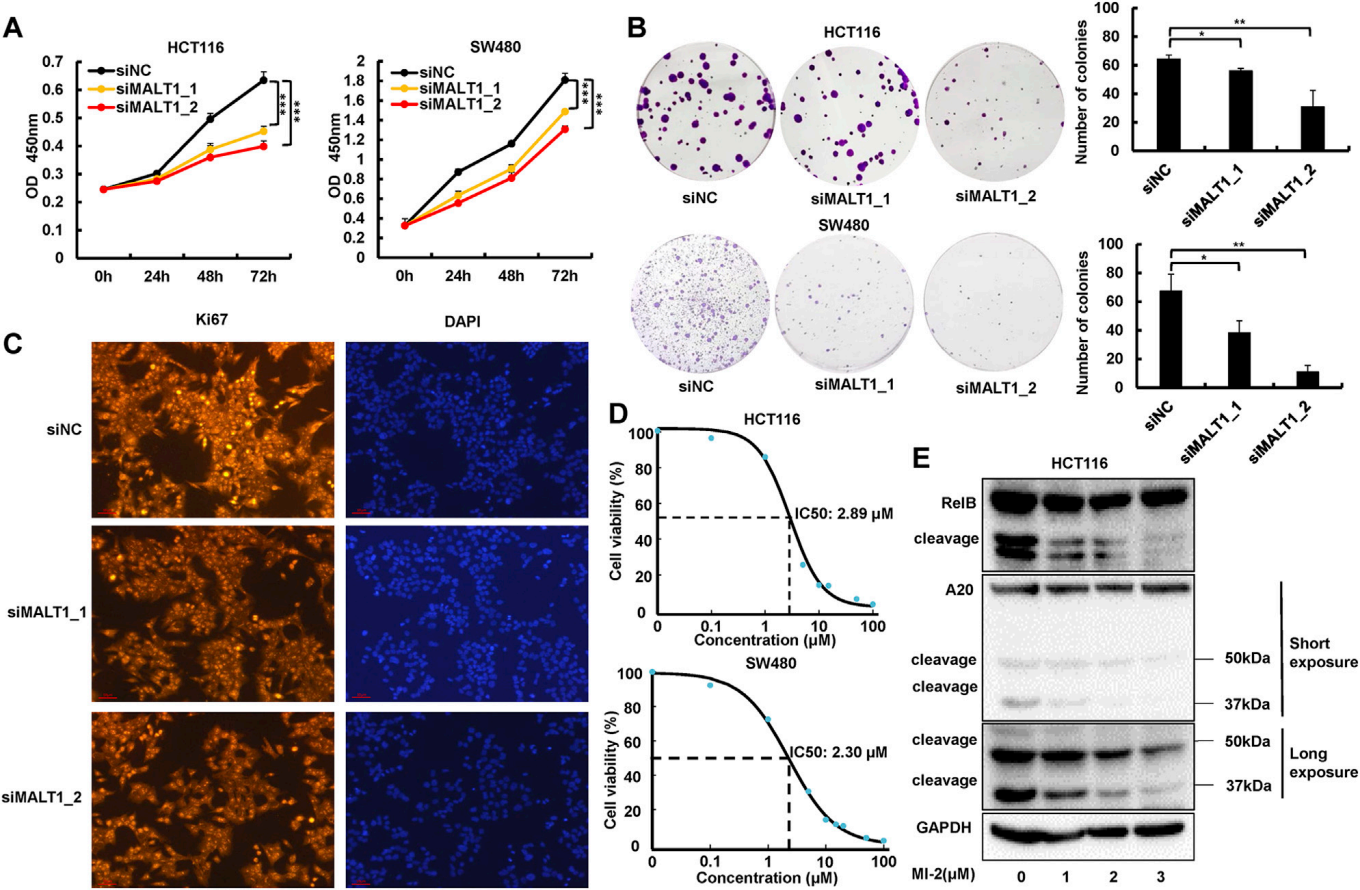
Inhibition of MALT1 reduced CRC cell proliferation. **(A)** Cell proliferation of CRC cells was determined by CCK-8 assays at 24, 48, and 72 h after transfection, as described. **(B)** Colony formation was assessed by crystal violet staining in CRC cells after transfection. **(C)** Proliferation indicator Ki67 was determined by immunofluorescence (IF) staining in HCT116 cell. **(D)** IC_50_ of MI-2 in HCT116 and SW480 cells was detected by CCK-8 assays. **(E)** MALT1 cleavage of RelB and A20 inhibition by MI-2 in HCT116 cells determined by immunoblotting. Each experiment was performed in triplicate, and data are presented as mean ± SD. One-way ANOVA and Dunnett’s multiple comparison test were used to analyze the data (*p < 0.05, **p < 0.01, ***p < 0.001).

In order to identify the role of MALT1 in CRC malignancy, we transiently transfected HCT116 or SW480 cells by siRNA and an expression plasmid, and the protein levels of MALT1 were determined by immunoblotting (Supplementary Figures S1A,B). The CCK8 assays were conducted, and the results revealed a significant decrease of absorbance in HCT116 or SW480 cells following MALT1 knockdown, compared with the control group (Figure 2A). As shown in Figures 2B,C, silencing the expression of MALT1 also decreased the number of colonies and the staining intensity of the proliferation marker Ki67. A small molecule inhibitor MI-2 was selected to inhibit MALT1 protease activity irreversibly. The cell proliferation of HCT116 and SW480 cells treated with MI-2 was significantly inhibited compared with control (Figure 2D). Then, a dose-dependent decrease of cleaved RelB or A20 was induced by MI-2, verifying its inhibitory effect (Figure 2E). When MALT1 was upregulated in CRC cells, the results were opposite with the silence of MALT1 (Supplementary Figures S1C–E). These data above suggested that MALT1 could promote the cell proliferation of CRC cells *in vitro*.

### MALT1 Inhibition Impairs Migration Ability of CRC Cells

**FIGURE 3:**
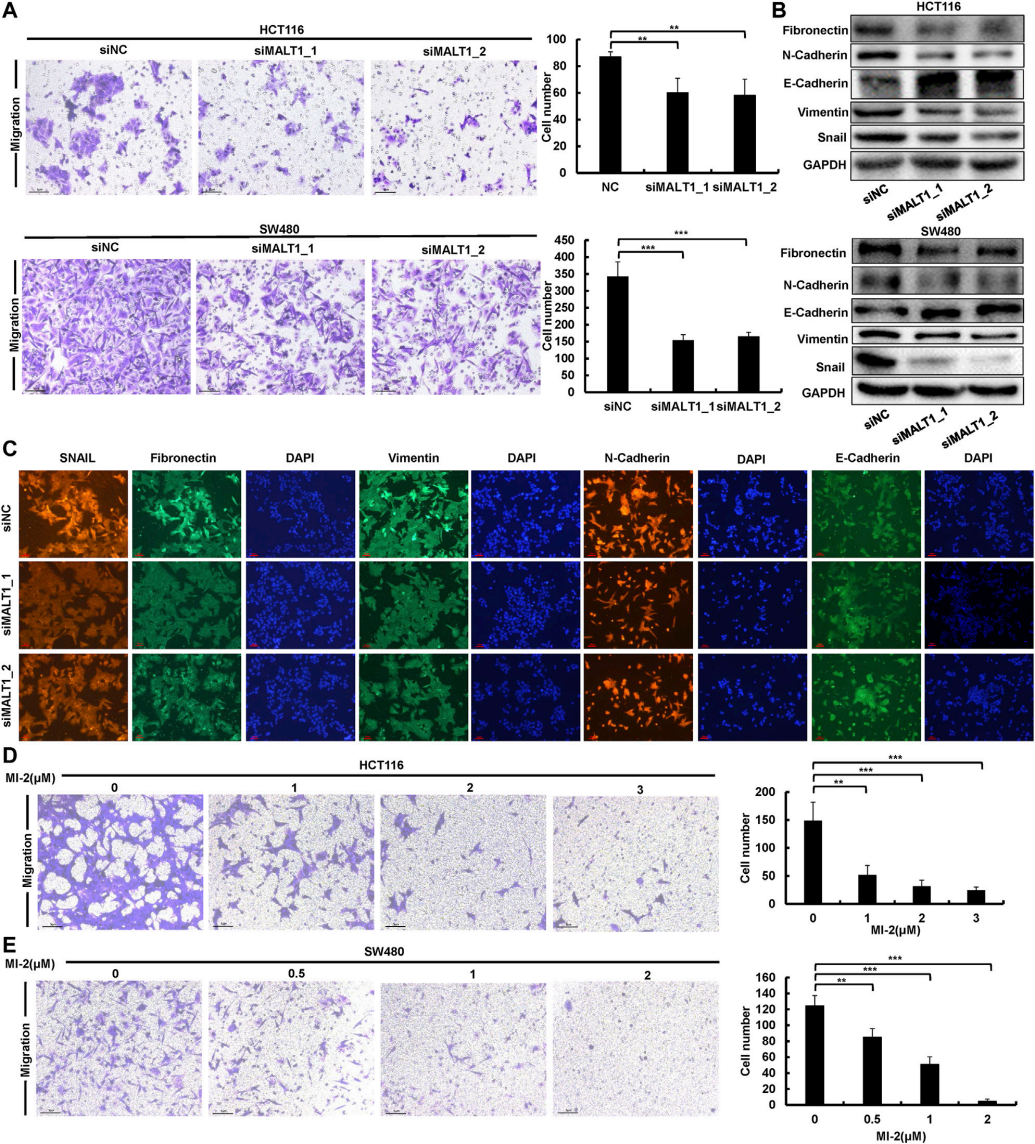
Downregulation of MALT1 inhibited CRC cell migration. **(A)** Cell migration of CRC cells transfected, as described, was determined by transwell assays. **(B)** EMT-associated protein level was examined by immunoblotting in CRC cells after transfection. **(C)** The expression of EMT-associated protein was determined by IF in HCT116 cell after transfection. MI-2 inhibited HCT116 **(D)** and SW480 **(E)** cell migration was detected by transwell assays. Each experiment was performed in triplicate and data are presented as mean ± SD. One-way ANOVA and Dunnett’s multiple comparison test were used to analyze the data (*p < 0.05, **p < 0.01, ***p < 0.001).

Metastasis is a vital hallmark of carcinomas progressing to higher pathological grades of malignancy. Therefore, to further investigate the effect of MALT1 on the malignancy of CRC cells, we performed transwell assays to test the migration ability of CRC cells after transfection. The results showed that the migration abilities in both HCT116 and SW480 cells were significantly suppressed after MALT1 was silenced by siRNA transfection (Figure 3A). In addition, the expression of EMT-related proteins, which were indicators of tumor metastasis, were detected by immunoblotting. As shown in Figure 3B, the knockdown of MALT1 resulted in decreased expressions of vimentin, fibronectin, Snail, and N-cadherin and the increased expression of E-cadherin. Meanwhile, we examined the expression of EMT-associated proteins by IF, and the results were consistent with those by immunoblotting (Figure 3C). Similar to the knockdown of MALT1, MI-2 treatment inhibited CRC cell migration in a dose-dependent manner (Figures 3D,E). Inversely, overexpressed MALT1 promoted cell migration in CRC cells (Supplementary Figures S2A–C). Generally, the data mentioned above demonstrated that MALT1 strongly enhanced the migration ability of CRC cells to promote the malignant phenotype.

### Loss of MALT1 Inhibits the Growth of Tumor *In Vivo*

**FIGURE 4:**
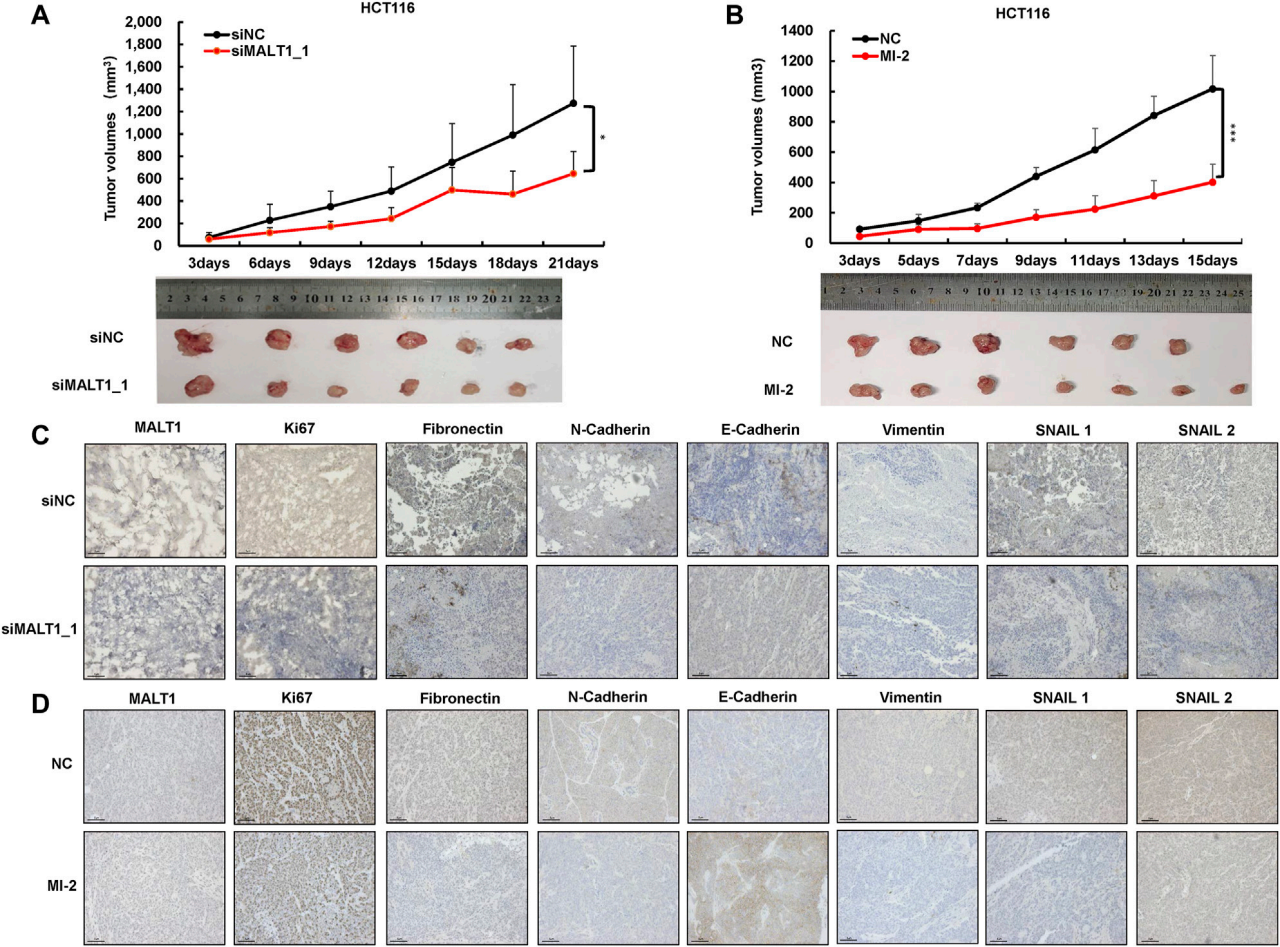
Knockdown of MALT1 inhibited CRC progression *in vivo*. **(A)** HCT116 cell transfected with siMALT1_1 was used to established subcutaneous tumor growth in a mouse xenograft model. Growth curves of tumor volumes were determined every 3 days. Representative photographs of tumors were shown below. **(B)** HCT116 cell was used to establish subcutaneous mouse model. After 3 days, mice were randomly divided into NC (5% DMSO) and MI-2 (25 mg/kg) group. Growth curves of tumor volumes were determined every 3 days. Representative photographs of tumors were shown below. **(C,D)** Representative IHC images of MALT1, Ki67, and on EMT-associated proteins’ tumor sections. Each experiment was performed in triplicate, and data are presented as mean ± SD. One-way ANOVA and Dunnett’s multiple comparison test were used to analyze the data (*p < 0.05, **p < 0.01, ***p < 0.001).

To evaluate the pro-oncogenic function of MALT1 *in vivo*, we established a subcutaneous tumor model. HCT116 cells were transfected with two different siRNAs targeting MALT1, and 1 × 10^7^ cells with MALT1 knockdown or negative control were injected subcutaneously into the two flanks of nude mice, respectively. Compared with the negative control group, the tumor volumes in the MALT1 knockdown group were significantly decreased in both two siRNAs (Figure 4A and Supplementary Figure S3A). Tumors were removed 21 days after inoculation. Then, the results showed that the MALT1 expression level, detected by IHC, was significantly decreased in the MALT1 knockdown group (Figure 4C and Supplementary Figure S3B). In accordance with our *in vitro* observations, the staining intensity of the proliferation marker Ki67 was much lower in xenografts derived from the MALT1 knockdown group than those in the control group (Figure 4C and Supplementary Figure 3B). As for the migration indicator, we performed IHC assays to analyze the expression of EMT-associated proteins, and the results were similar to *in vitro* results (Figure 4C and Supplementary Figure S3C).

Meanwhile, HCT116 cells were injected subcutaneously to identify the effect of MI-2 *in vivo*. Three days after injection, mice were randomly divided into two groups, the DMSO and MI-2 groups. The results showed that the tumor volumes in the MI-2 group were significantly reduced compared to the DMSO group (Figure 4B). Consistently, the expression of Ki67 and EMT-associated proteins was lower in MI-2 treated mice than that in DMSO (Figure 4D). Taken together, these results showed that the inhibition of MALT1 or its protease activity could suppress tumor growth and migration *in vivo*.

### MALT1 Activates NF-κB Signaling Pathway in CRC Cells

**FIGURE 5:**
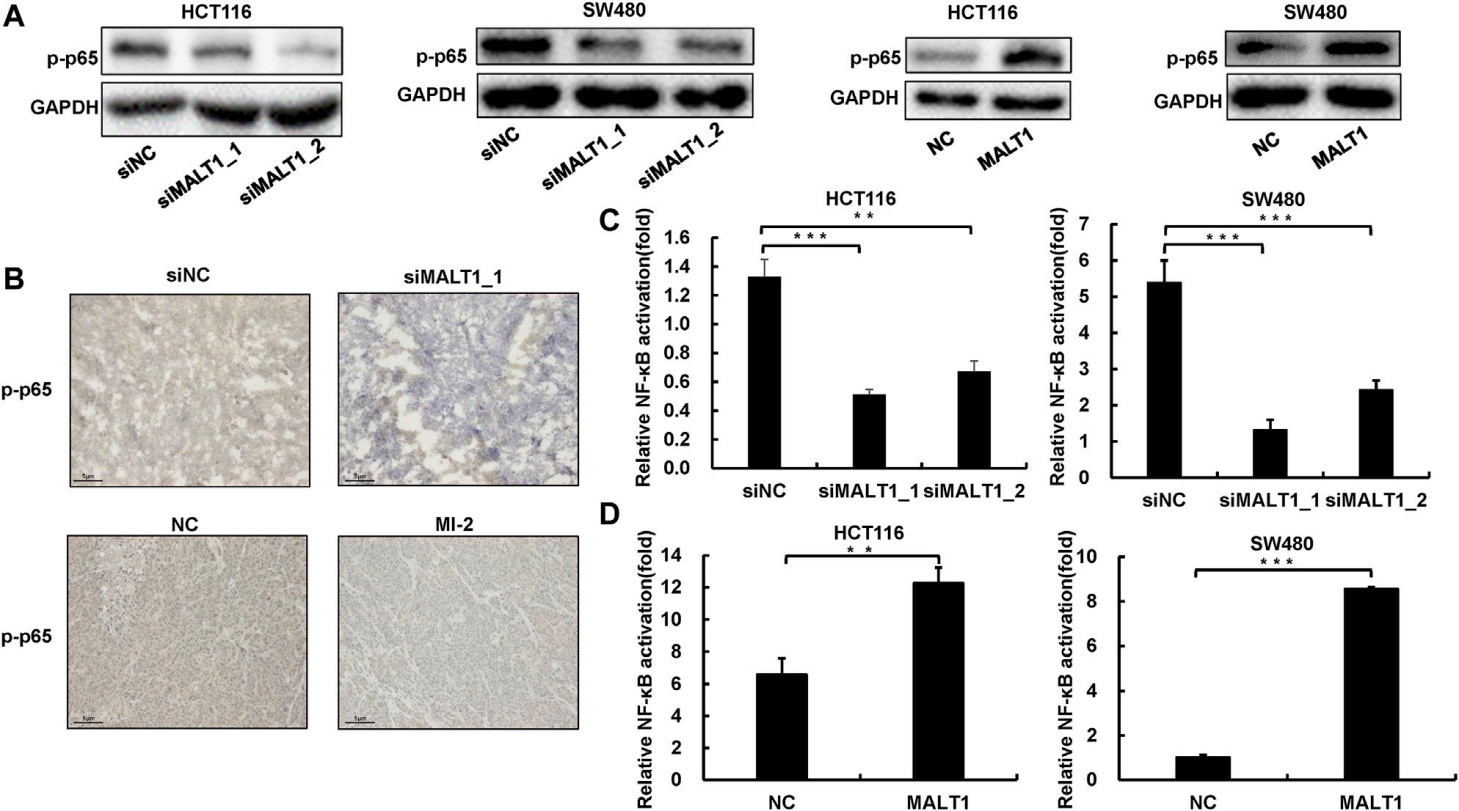
Loss of MALT1 impaired NF-κB pathway activation. **(A)** The protein level of p-p65 in CRC cells transfected, as described, was determined by immunoblotting. **(B)** Representative IHC images of p-p65 on tumor sections. Dual-luciferase reporter assays were used to analyze NF-κB activation in CRC cells **(C,D)**. Each experiment was performed in triplicate, and data are presented as mean ± SD. One-way ANOVA and Dunnett’s multiple comparison test were used to analyze the data (*p < 0.05, **p < 0.01, ***p < 0.001).

To investigate the downstream molecular mechanism of MALT1 promoting CRC malignancy, we detected the activation of the NF-κB pathway, which has been reported as the downstream. The phosphorylation of p65 is a hallmark of NF-κB activation, which results in p65 nuclear translocation and further transcriptionally activates the target genes. Given that, we examined the expression of phosphorylated p65 by immunoblotting, and the results showed that the downregulation of MALT1 inhibited the phosphorylation of p65, whereas the upregulation of MALT1 increased it (Figure 5A). *In vivo* experiments also demonstrated that MALT1 knockdown or its protease activity inhibition led to a decrease of phosphorylated p65 in mice subcutaneous tumor tissues (Figure 5B and Supplementary Figure S3B). To verify the activation of the NF-κB pathway, luciferase reporter assays were also performed. As shown in Figures 5C,D, the luciferase activity of the NF-κB reporter was 4–6 times in the negative control group that of the inhibition of the MALT1 group, and overexpressed MALT1 increased the transcriptional activity compared with the control group. According to our data, MALT1 could promote CRC development by targeting the NF-κB signaling pathway (Figure 5E).

### miR-365a-3p and miR-375 inhibit NF-κB Signaling Pathway *via* Targeting MALT1

**FIGURE 6:**
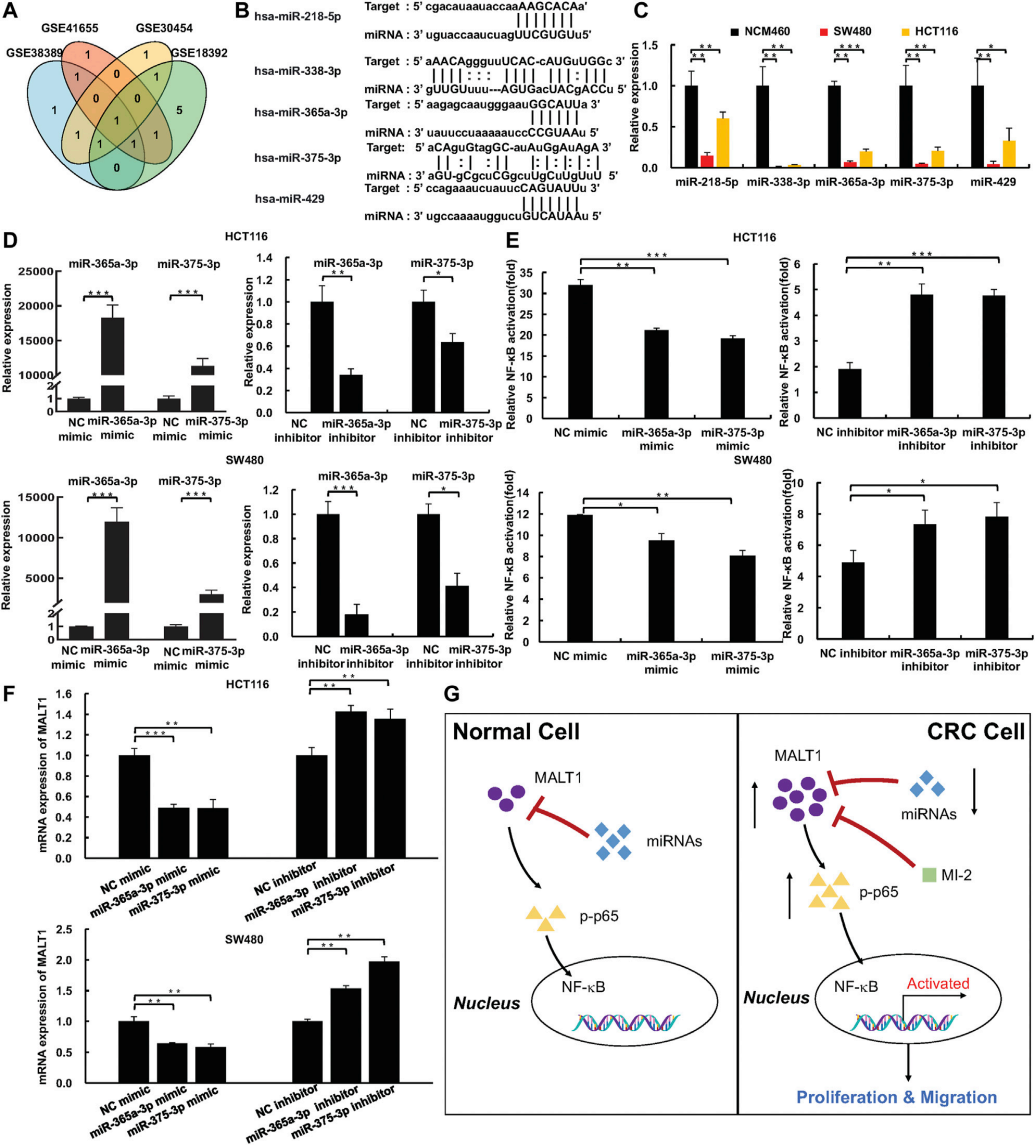
miR-375 and miR-365a-3p inhibited NF-κB pathway activation *via* targeting MALT1. **(A)** miR-375, miR-365a-3p, miR-429, miR-218, and miR-338-3p targeted MALT1 predicted by miRcode and were downregulated in CRC based on GEO database. **(B)** miR-375, miR-365a-3p, miR-429, miR-218, and miR-338-3p binding sites within 3′UTR of MALT1 mRNA. **(C)** The expression of miR-375, miR-365a-3p, miR-429, miR-218, and miR-338-3p in NCM and CRC cell lines detected by qPCR. **(D)** The expression of miR-375 and miR-365a-3p in CRC cells transfected with miRNA mimic or miRNA inhibitor. **(E)** Dual-luciferase reporter assays were used to analyze NF-κB activation in CRC cells. **(F)** The mRNA level of MALT1 detected by qPCR in CRC cells after transfection as described. **(G)** Simplified model of MALT1 promoting CRC development *via* miR-375/miR-365a-3p/NF-κB pathway. Each experiment was performed in triplicate, and data are presented as mean ± SD. One-way ANOVA and Dunnett’s multiple comparison test were used to analyze the data (*p < 0.05, **p < 0.01, ***p < 0.001).

To gain a better understanding of the complete regulatory axis of MALT1-NF-κB, miRcode and GEO databases were carried out to identify a number of miRNAs targeting MALT1. Through miRcode database prediction, 97 miRNAs probably targeted MALT1 (Supplementary Table S1). Among these miRNAs, downregulation miRNAs in CRC tissues compared with normal tissues were sorted out based on GEO databases (GSE18392, GSE38389, GSE41655, and GSE30454, Supplementary Table S2). We chose miR-375, miR-365a-3p, miR-338-3p, miR-218, and miR-429 for further exploration, which was included in more than two GEO databases (Figures 6A,B). Then, real-time PCR experiments were exerted to detect the relative RNA expression of each miRNA in NCM and CRC cells, and the results showed that all those expressions were higher in NCM cells as expected (Figure 6C). Since MALT1 was targeting the NF-κB pathway to increase the malignancy of CRC, miR-365a-3p and miR-375 were selected as reported in previous studies (Sun et al., 2018; Yin et al., 2019). Through the luciferase reporter assays, the results confirmed that the overexpression of miR-365a-3p or miR-375 inhibited NF-κB activation, which is in accordance with the results of MALT1 inhibition, and the downregulation of miR-365a-3p or miR-375 promoted NF-κB activation (Figures 6D,E). Furthermore, the mRNA expression of MALT1 regulated by miR-365a-3p or miR-375 was detected by qPCR. As expected, MALT1 expression was significantly decreased after the miR-365a-3p or miR-375 mimic treatment and increased after the miR-365a-3p or miR-375 inhibitor treatment (Figure 6F). In conclusion, miR-365a-3p or miR-375 might target MALT1 to inhibit NF-κB activation in CRC cells (Figure 6G).

## Discussion

CRC is the third commonly diagnosed cancer and the world’s second most deadly cancer in 2020 (Sung et al., 2021). Although there are many treatments including endoscopic and surgical local excision, targeted therapy, and immunotherapy, the morbidity and mortality of CRC are still rising steadily every year (Dekker et al., 2019; Siegel et al., 2020). MALT1 has been found have a vital role in lymphoma and immune cell development in the last two decades. The loss of MALT1 contributes to infantile combined immunodeficiency and immune dysregulation coupled with lymphocyte signaling impaired *via* the NF-κB pathway (Punwani et al., 2015). Indeed, MALT1 promotes cancer malignant progression not only in lymphoma but also in other types of cancer, such as melanoma and lung cancer (Pan et al., 2016; Rosenbaum et al., 2019). Here, in our study, we have firstly verified that MALT1 was upregulated in CRC, and the expression MALT1 was negatively correlated with the prognosis of CRC. Also, we verified that MALT1 promotes the cell proliferation and migration of CRC with the activation of the NF-κB pathway.

NF-κB has been found over 30 years as a rapidly inducible transcription factor, which has been confirmed a crucial role in tumor malignancy. NF-κB is directly bound to the receptors of TNF or IL-1, leading to the upregulation of anti-apoptosis genes, such as BCL-XL (Karin and Greten, 2005). Besides, the transcriptions of SLUG, TWIST1, and SNAIL were also stimulated by NF-κB, which initiated EMT and augmented cell migratory behavior (Taniguchi and Karin, 2018). In CRC, a number of studies have confirmed that some molecules, such as MyD88, SREBP1, and CXCL5, promoted CRC cell proliferation, migration, invasion, and tumor growth through NF-κB activation (Chen et al., 2019; Gao et al., 2019; Zhu et al., 2020). In addition, there were biological crosstalks between the NF-κB pathway and other signaling pathways, such as the EGFR pathway (Wang et al., 2019). NF-κB inhibitors have been exploited and studied for a long time, mainly proteasome blockers and IKK inhibitors. Bortezomib, the best studied proteasome inhibitor of NF-κB, reduced over 50% of patients with resistance to the therapy in a phase 2 study (Richardson et al., 2003). NSAIDs, mainly targeting COX2, which is regulated by NF-κB signaling, are clinically used for preventing the recurrence of CRC (Katona and Weiss, 2020). In spite of their widespread applications, side effects are still in company with the inhibitors for CRC treatment, such as neutrophilia, fever, and abnormal IL-1 release (Zhang et al., 2017). Thus, exploring new targeted agents seems to be a long-term and beneficial strategy.

It is explicit that MALT1 activates the NF-κB pathway through the CBM complex in lymphoma. After the activation of TCR or BCR, CARMA1 is recruited to the plasma membrane, followed by BCL10 and MALT1; then, the CBM complex is assembled. This complex activates the IKK complex through ubiquitination, which further phosphorylates IκB, and its degradation activates the NF-κB pathway. MI-2 is a small molecule inhibitor that directly binds to MALT1 and suppresses the protease function, which is irreversible (Fontan et al., 2012). DSS-induced colitis reduced the diversity of the intestinal microbiome of mice, which was reversed by MI-2. MI-2 also reshaped the host’s immune-modulating capacity by reducing inflammatory cytokines, such as TNF-α, IL-1β, IL-17α, and IL-22 (Lee et al., 2018). In addition, the activation of the NLRP3 inflammasome was also inhibited by MI-2 (Liu et al., 2016). The above researches suggest a positive role of MALT1 or its protease function at least in the treatment of colorectal diseases. Previous studies proved that MI-2 inhibited ABC-DLBCL and melanoma tumor growth *in vivo* and inhibited MALT1 proteolytic activity and NF-κB activation *in vitro* (Fontan et al., 2012; Saba et al., 2017; Di Pilato et al., 2019). In our study, MI-2-treated CRC cells had dampened migration capability *in vitro*, which put up a promising target agent for CRC.

Nowadays, more and more miRNAs have been identified as biomarkers and potential therapeutic targets of cancers. The five miRNAs in our study have also been identified as crucial mediators of CRC, and all of them were downregulated in CRC, while only miR-365a-3p and miR-375 have been confirmed to directly suppress NF-κB activation (Sun et al., 2018; Yin et al., 2019). miR-365a-3p expression was associated with the overall survival of CRC patients and inhibited CRC cell invasion, migration, and chemoresistance through targeting KLF3 (Hong et al., 2020; Li et al., 2021). Plenty of literature verified that miR-375 was downregulated in CRC and suppressed the malignant progression of CRC (Wang et al., 2014; Xu et al., 2019). Moreover, miR-375 enhanced the chemosensitivity of CRC to 5-fluorouracil, which provides a hopeful therapeutic strategy (Xu et al., 2020). Some researches considered miR-429 as a biomarker for CRC, which had lower expression in CRC patients with poor prognosis, and proved that miR-429 inhibited the cell proliferation and migration of CRC (Sun et al., 2014a; Sun et al., 2014b; Guo et al., 2020). miR-218 also plays a pivotal role in CRC malignancy, including being associated with poor prognosis in patients and suppressing EMT and angiogenesis (Yu et al., 2013; Lun et al., 2018). In accordance with the other four miRNAs, miR-338-3p also inhibited CRC cell growth and migration and conferred 5-fluorouracil resistance in p53 mutant CRC (Han et al., 2017; Lu et al., 2019). Besides identifying the downregulation of five miRNAs in CRC, our study also proved that miR-375 and miR-365a-3p inhibited the NF-κB pathway *via* targeting MALT1, which provided a new insight of CRC therapy.

Taken together, our study demonstrated that MALT1 promotes CRC progression *via* NF-κB activation. The inhibition of MALT1 proteolytic function or silencing its expression suppressed the proliferation and metastasis of CRC cells through NF-κB activation. Moreover, miR-375 and miR-365a-3p inhibited NF-κB activation by targeting MALT1 in CRC. Our research unveiled that MALT1 acted as an oncogene in the progression of CRC and provided a novel therapeutic target for CRC clinical treatment.

## Data Availability

The original contributions presented in the study are included in the article/Supplementary Material, further inquiries can be directed to the corresponding authors.
