# Supplementary figures and images for: Targeting MALT1 Suppresses the Malignant Progression of Colorectal Cancer via miR-375/miR-365a-3p/NF-κB Axis
Source: Front Cell Dev Biol. 2022 Mar 2;10:845048. doi: 10.3389/fcell.2022.845048 (PMC8924071; doi:10.3389/fcell.2022.845048)

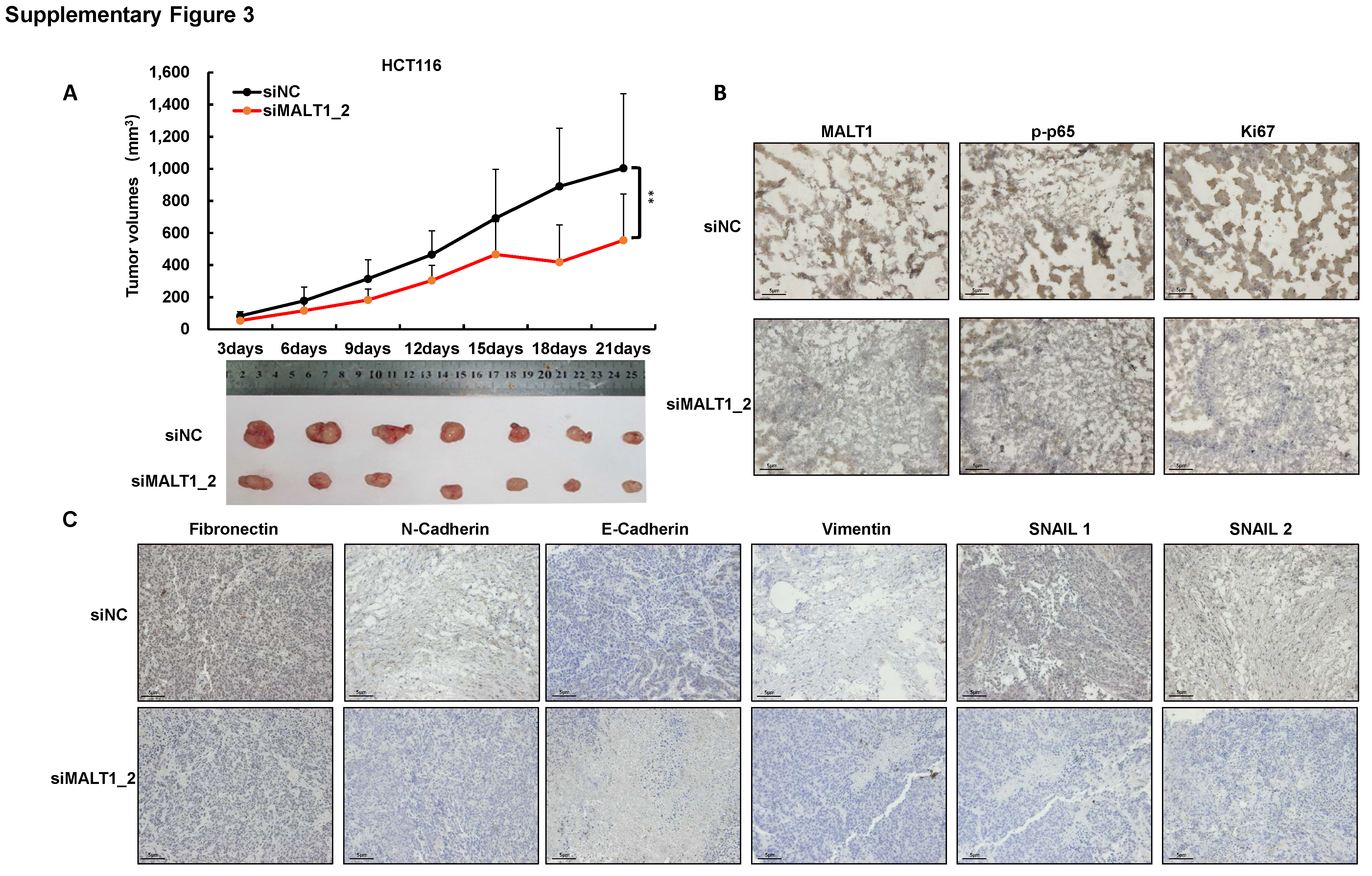

Supplement: Supplementary file 1 [file Image3.TIFF]

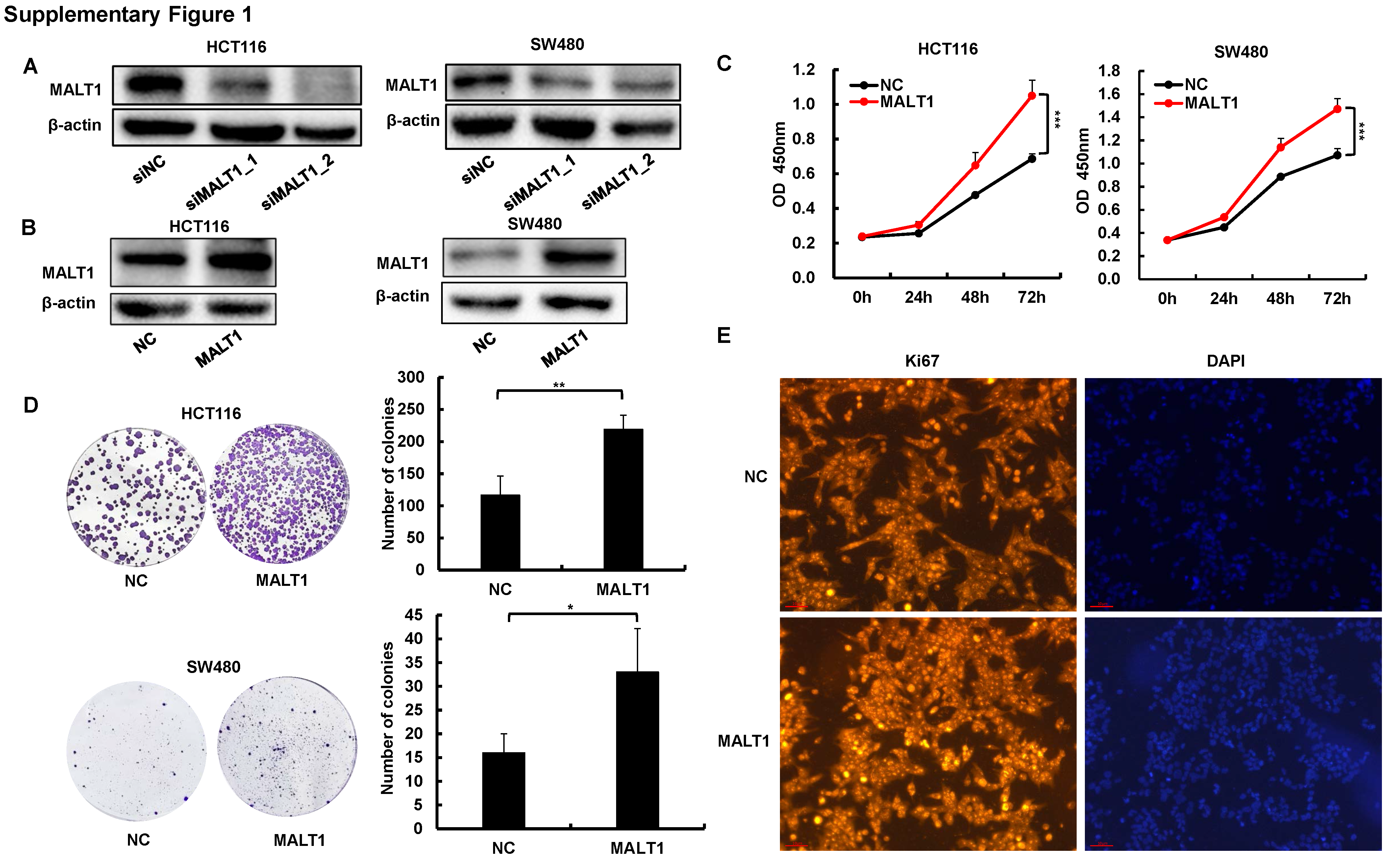

Supplement: Supplementary file 3 [file Image1.TIFF]

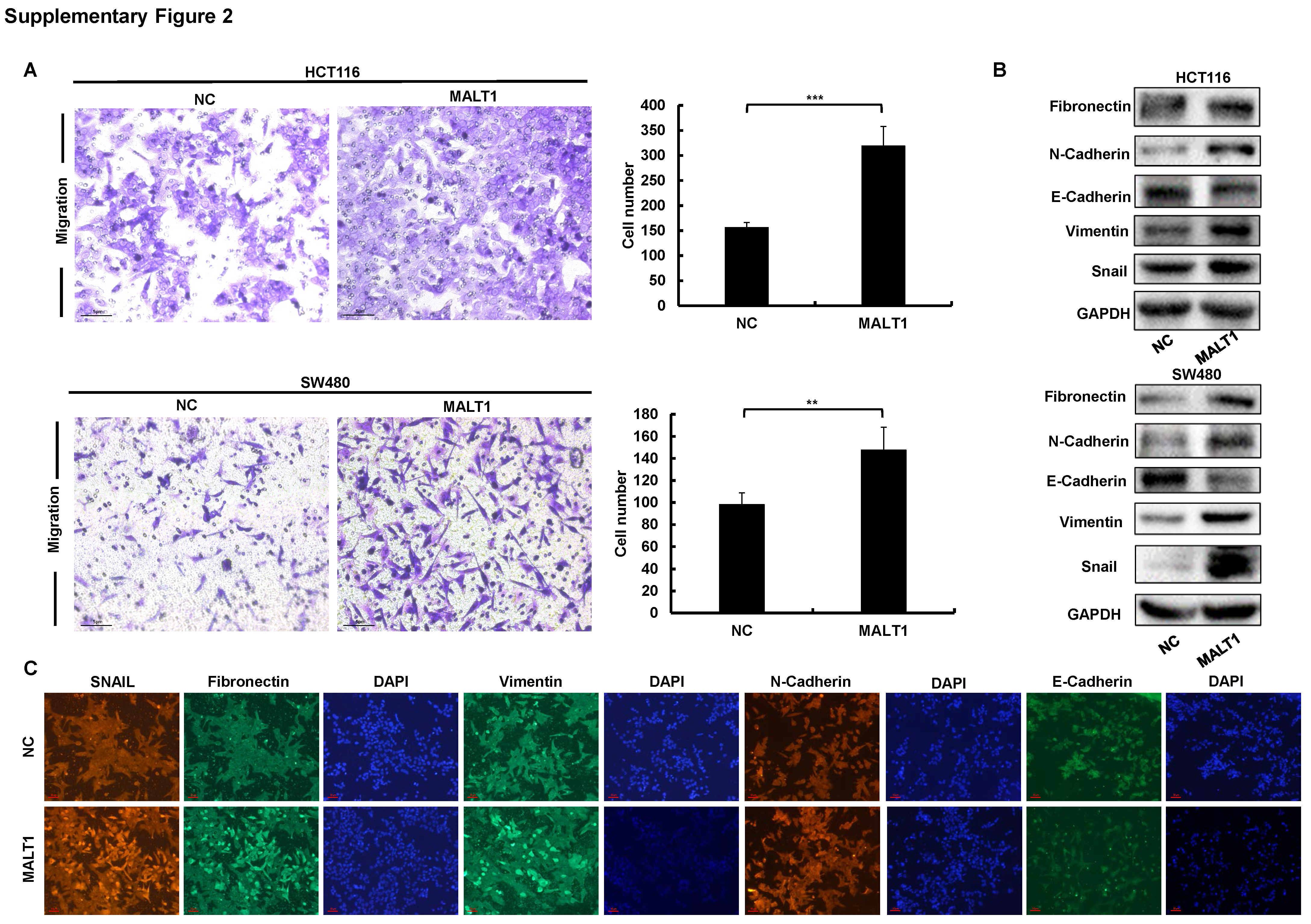

Supplement: Supplementary file 5 [file Image2.TIFF]
